# Experience-Related Structural Changes of Degenerated Occipital White Matter in Late-Blind Humans – A Diffusion Tensor Imaging Study

**DOI:** 10.1371/journal.pone.0122863

**Published:** 2015-04-01

**Authors:** Susanne Dietrich, Ingo Hertrich, Vinod Kumar, Hermann Ackermann

**Affiliations:** 1 Department of General Neurology—Center for Neurology, Hertie Institute for Clinical Brain Research, University of Tübingen, Hoppe-Seyler-Str. 3, D-72076, Tübingen, Germany; 2 Department of Psychiatry, Psychotherapy and Psychosomatics, University Hospital, RWTH, Aachen University, Aachen, Germany; University of Pennsylvania, UNITED STATES

## Abstract

Late-blind humans can learn to understand speech at ultra-fast syllable rates (ca. 20 syllables/s), a capability associated with hemodynamic activation of the central-visual system. Thus, the observed functional cross-modal recruitment of occipital cortex might facilitate ultra-fast speech processing in these individuals. To further elucidate the structural prerequisites of this skill, diffusion tensor imaging (DTI) was conducted in late-blind subjects differing in their capability of understanding ultra-fast speech. Fractional anisotropy (FA) was determined as a quantitative measure of the directionality of water diffusion, indicating fiber tract characteristics that might be influenced by blindness as well as the acquired perceptual skills. Analysis of the diffusion images revealed reduced FA in late-blind individuals relative to sighted controls at the level of the optic radiations at either side and the right-hemisphere dorsal thalamus (pulvinar). Moreover, late-blind subjects showed significant positive correlations between FA and the capacity of ultra-fast speech comprehension within right-hemisphere optic radiation and thalamus. Thus, experience-related structural alterations occurred in late-blind individuals within visual pathways that, presumably, are linked to higher order frontal language areas.

## Introduction

It is well established that early-blind individuals, i.e., people with congenital blindness or loss of sight soon after birth, show structural changes of the pathways and relay stations of the cerebral visual system [1–5]. Even in case of a later onset, vision loss appears to have an impact upon morphological features of the brain. Thus, recent diffusion tensor imaging (DTI) studies [6–7] revealed white matter alterations in late-blind subjects (loss of sight after the age of five years). Furthermore, voxel-based morphometric techniques documented atrophy (volume reduction) of, e.g., the primary visual cortices (including BA17/18) as well as the optic chiasm and radiations both in early- and late-blind subjects (early-blind: [4, 8–10]; late-blind: [11–13]). And structural measures appear to predict pitch and melody discrimination abilities at least in early-blind subjects [14–15]. Such morphological changes likely reflect neural degenerative processes subsequent to loss of afferent input and/or the consequences of altered visual experience during sensitive periods of brain development [1]. Besides blindness-induced structural alterations, vision loss may give rise to cross-modal reorganization of the visual system. For instance, functional magnetic resonance imaging (fMRI) studies found task-related visual cortex activation in early- and late-blind persons—with stronger effects in case of an early disorder [16–19]—in response to somatosensory [16, 19, 20–24] and auditory stimuli [25–28] or during engagement in higher cognitive tasks [29–34]. Under these conditions, early- and late-blind subjects differ not only in the size of activation clusters [16–17, 30, 35–37], but also in the distribution of activation clusters of the visual system. For example, subsequent to vision loss early in life, visual regions may be used for similar language-related computations in a non-visual modality [38] as well as auditory spatial/motion processing [37, 39]. Furthermore, transcranial magnetic stimulation (TMS) provided some evidence for a functionally relevant recruitment of the occipital cortex in early-blind humans during non-visual tasks [40–42]. Most noteworthy, a TMS study by Cohen and colleagues [36] was able to document that transient functional disruption of the occipital cortex hampers Braille reading in congenital and early-blind subjects, but not in late-onset blind individuals. As another example of cross-modal interactions after vision loss, early- and late-blind individuals can learn to manage—by repeated exposure to accelerated verbal utterances using, e.g., computer screen reading systems—to comprehend spoken language at enhanced speaking rates of up to 22 syllables per second (syl/s), an accomplishment exceeding by far the limits of untrained subjects (ca. 8 syl/s) [43]. As compared to early-blind subjects, neuroplasticity of the visual system has been studied, however, far less extensively in individuals with blindness of a later onset [44]. A previous fMRI investigation of our group [45] found in late-blind subjects during application of ultra-fast speech materials—besides hemodynamic activation of the “classical” perisylvian language zones (left-hemisphere inferior frontal gyrus, bilateral superior/middle temporal gyrus/sulcus)—covariation of the blood oxygen level-dependent (BOLD) responses of right-hemisphere primary visual cortex (V1), bilateral pulvinar (Pv), and the anterior part of the left-hemisphere supplementary motor area (pre-SMA) with the level of ultra-fast speech comprehension capabilities (16 syl/s). In order to further elucidate the structural underpinnings of this feat, DTI was performed in late-blind individuals known to show task-related hemodynamic activation of the visual cortex—as determined by means of functional magnetic resonance imaging (fMRI)—during an ultra-fast speech comprehension task (listening to time-compressed verbal utterances) [45–46]. Against the background of our preceding studies, we expected, first, blindness-induced structural degeneration of white matter tracts pertaining to the visual system, especially, the optic radiation—compared to a group of normally sighted subjects serving as a baseline condition. Second, blind subjects with low and high ultra-fast speech comprehension capabilities should differ in the structural properties of the white matter components of the visual system. For example, high-performing late-blind individuals may show preserved or revitalized visual pathways by contrast to patients with low perceptual skills. Based on our previous investigations [45], third, the expected skill-related structural changes must be assumed to predominantly affect right-hemisphere visual components and ipsilateral regions close to Pv in late-blind individuals. Although other linguistic tasks, e.g., verb generation, lexical access, and sentence combinatorial tasks, primarily activated left-hemisphere occipital cortex both in congenitally/early- and late-blind participants [29–30, 38, 47], our preceding studies of ultra-fast speech perception [45, 48] consistently documented a predominant contribution of right-hemisphere occipital cortex to the comprehension of ultra-fast speech materials. It was assumed [49], based upon these observations, that the visual cortex of this side contributes to the generation of syllabic trigger signals forwarded to the anterior perisylvian areas of the left hemisphere, compensating for the inability of the central-auditory system to separate syllable-prosodic from segmental information (consonants, vowels) at ultra-fast speech rates exceeding 16 syl/s. More specifically, late-blind subjects seem to be able to “open” a further information channel projecting from the auditory pathway via superior colliculus and Pv to primary visual cortex (secondary visual pathway) [49].

In order to test these three hypotheses, a group of late-blind subjects (onset of blindness > 5 years) as well as normally sighted participants serving as a clinical control sample (by and large, the same subjects who had participated in a preceding fMRI study of our group [45]) underwent DTI, a well-established approach to structural magnetic resonance imaging (MRI) of white matter structures [50]. Briefly, the “tensors” reflecting the “direction of movement” of water molecules were determined at the level of individual voxels, and these parameters then allowed for the calculation of fractional anisotropy (FA) as a quantitative measure of the directionality of water diffusion [51–53]. High FA values point at the presence of directional diffusion (anisotropy) in fiber tracts whereas low FA values (isotropy) are usually associated with the cellular structures lacking such a parallel geometry (diffusion ellipsoid), i.e., gray matter or regions of crossing fiber tracts. Decreased FA values, furthermore, may indicate a loss of myelination and/or axonal organization [54]. Thus, FA values provide a parameter of local tissue geometry such as, e.g., the degree of fiber myelination which may be subject to neuroplastic changes induced by sensory deprivation (blindness), on the one hand, and by the acquisition of distinct skills (e.g., ultra-fast speech comprehension), on the other [55–56].

## Materials and Methods

### Participants

A total of fifteen late-blind individuals (13 males; mean age = 40.73 years, *SD* = ± 13.83; vision loss after the age of 5 years; mean age of blindness onset = 24.13 years, *SD* = 16.65) fourteen normally sighted subjects (9 males; mean age = 37.93 years, *SD* = ± 12.8), and a normally sighted subject with extensive training in ultra-fast speech comprehension (female, age = 23 years) participated in the DTI experiment (Table 1). All of them were right-handed (Edinburgh handedness inventory) native German speakers without a history of neurological diseases or hearing deficits (determined by means of an audiogram). The study design had been approved by the ethics committee of the University of Tübingen, and written informed consent was obtained prior to the structural MRI measurements from all subjects. Since the late-blind subjects had been recruited from community organizations, information on the etiology and time course of the ophthalmological disorders had to be garnered from personal interviews and clinical reports provided by the participants. In all instances, a peripheral origin of blindness could be established. The participants represented, however, a rather heterogeneous group with respect to their age at the onset of vision loss (Table 1). Furthermore, some of the late-blind subjects had minor residual visual capabilities such as light and color sensitivity, but all of them showed no or—if at all—highly diminished visual acuity. Visual acuity was determined by a pattern discrimination task in terms of minimal distance of recognition of two points divided by minutes of arc (ICD-9-CM, International Classification of Diseases, Ninths Revision, Clinical Modification, http://www.cdc.gov/nchs/icd/icd9cm.htm). The present group of late-blind participants comprised 12 subjects with a visual acuity of 0.01 (*n* = 2) or less (*n* = 10), i.e., almost total blindness, and three subjects with profound vision loss (visual acuity amounting to 0.02 or 0.03).

**Table 1:** Clinical and behavioral data of the vision-impaired and normally sighted subjects.

**Subjects**	**Performance of understanding ultra-fast speech (%)**	**Age of blindness onset (years)**	**Duration of blindness related to age (%)**	**Chrono-logical age (years)**	**Etiology of blindness**	**Characterization of visual deficits, in parentheses visual acuity** *
102	93	7	78	32	retinal detachment	no residual visual perception (0)
147	86	7	87	52	retinitis pigmentosa	no residual visual perception (0)
108	72	26	16	31	macular degeneration	light perception (0.01)
181	69	13	48	25	optic nerves damaged by a tumor	no residual visual perception (0)
104	67	7	65	20	hereditary vision loss (gene mutation)	no residual visual perception (0)
112	65	44	8	48	retinitis pigmentosa	light perception (0.01)
0Z4	64	37	14	43	glaucoma	no residual visual perception (0)
111	62	24	27	33	retinal damages	no residual visual perception (0)
113	60	17	58	40	uveitis intermedia	residual visual perception (0.03)
0Z9	57	13	52	27	retinal detachment	no residual visual perception (0)
105	39	18	54	39	retinal damages	no residual visual perception (0)
186	35	5	87	38	cataract	residual vision strongly reduced (0.02)
148	3	52	22	67	retinitis pigmentosa	no residual visual perception (0)
0Z5	0	47	8	51	eye cataract, glaucoma	residual vision strongly reduced (0.02)
109	0	45	31	65	retinal damages	no residual visual perception (0)
*142*	*88*	*-*	*-*	*23*	*n*.*s*.	
177	21	-	-	26	n.s.	
173	18	-	-	45	n.s.	
174	18	-	-	43	n.s.	
126	16	-	-	24	n.s.	
171	14	-	-	58	n.s.	
0Z0	11	-	-	27	n.s.	
021	9	-	-	44	n.s.	
130	8	-	-	24	n.s.	
0Y8	7	-	-	25	n.s.	
0Z6	6	-	-	25	n.s.	
127	6	-	-	27	n.s.	
176	6	-	-	40	n.s.	
101	5	-	-	31	n.s.	
0Z7	4	-	-	50	n.s.	

Italic number indicates a sighted individual who trained to understand ultra-fast speech.

* Visual acuity measures optic resolution in units of 1/minutes of arc, assessed at a distance of 1 m. Values below 0.01 were listed as zero. Abbreviations: n.s. = normally sighted

### Behavioral data acquisition and analyses

In order to obtain a quantitative behavioral measure of an individual’s capability to understand ultra-fast speech utterances, each subject performed a sentence repetition task based upon sentence utterances produced at an ultra-fast speaking rate (16 syl/s; screen reader software JAWS 2008, male voice, "eloquence" formant synthesizer, http://www.freedomsci.de, see [45]). The test materials (see [45] for audio-examples) were presented to the participants via loudspeakers (Fostex, Personal monitor, 6301B) within a sound-attenuated room. Subjects were asked to repeat them “as good as possible”, even when they had failed to “grasp” all the words of a stimulus. The spoken repetitions were digitally recorded (M-audio Microtrack 2496) and underwent subsequent assessment in terms of the determination of the percentage of correctly reproduced words (lexical items, irrespective of minor grammatical errors such as deviant singular or plural endings). Statistical evaluation of the behavioral data included non-parametric group comparisons (Mann-Whitney-U-test, late-blind versus sighted), after testing for normal distribution (Shapiro-Wilk-test), as well as correlation analyses (Pearson or Spearman tests), addressing the relationship between behavioral repetition performance, on the one hand, and age at the onset of vision loss, duration of blindness (related to the age of a participant), and age at the time of MRI measurements, on the other.

### Structural MRI data acquisition

DTI relied upon a 3 Tesla Siemens Trio MR system. Diffusion weighted images were acquired by means of a single-shot echo planar imaging (EPI) sequence aligned to the anterior-posterior commissural plane, using the following imaging parameters: 72 continuous axial slices with a thickness of 2 mm each, field of view = 260 mm, repetition time = 5500 ms, echo time = 95 ms, acquisition matrix = 128 x 128. The diffusion sensitizing gradients were applied along 35 non-collinear directions in order to calculate tensors (split into 3 diffusion directions given by the eigenvalues λ_1_, λ _2_, λ _3_) at the voxel-level each, providing a measure of diffusion along fibers in terms of FA values. In addition to the diffusion patterns, a reference image with no diffusion information was acquired, weighting the level of sensitivity to diffusion (quotient of diffusion and reference image). Thus, the amount of diffusion weighting has been measured by the b-value: b = 0 sec/mm^2^ indicates no diffusion weighting in case of the reference image; b = 1000 sec/mm^2^ was chosen for diffusion images.

### Data preprocessing and statistical evaluation

Correction for Eddy current distortions and motion artifacts in the DTI dataset relied upon affine alignment of each diffusion-weighted image (b = 1000 sec/mm^2^) to the reference image (b = 0 sec/mm^2^), using FMRIB’s diffusion toolbox (FSL, version 4.1.1, Eddy current correction). Subsequently, the diffusion tensors were fitted to the data, allowing for the determination of the principle diffusion directions (vectors) and the diffusion tensor eigenvalues (λ _1_, λ _2_, λ _3_), i.e., the strengths of diffusion in the three principle diffusion directions (FMRIB’s diffusion toolbox, DTIFIT reconstruct diffusion tensors). On this basis, thus, the FA value of each separate voxel could be calculated (expressed as a ratio between 0 and 1, where complete isotropic diffusivity is 0 and complete anisotropic diffusivity in a single direction amounts to 1).

**Fig 1:**
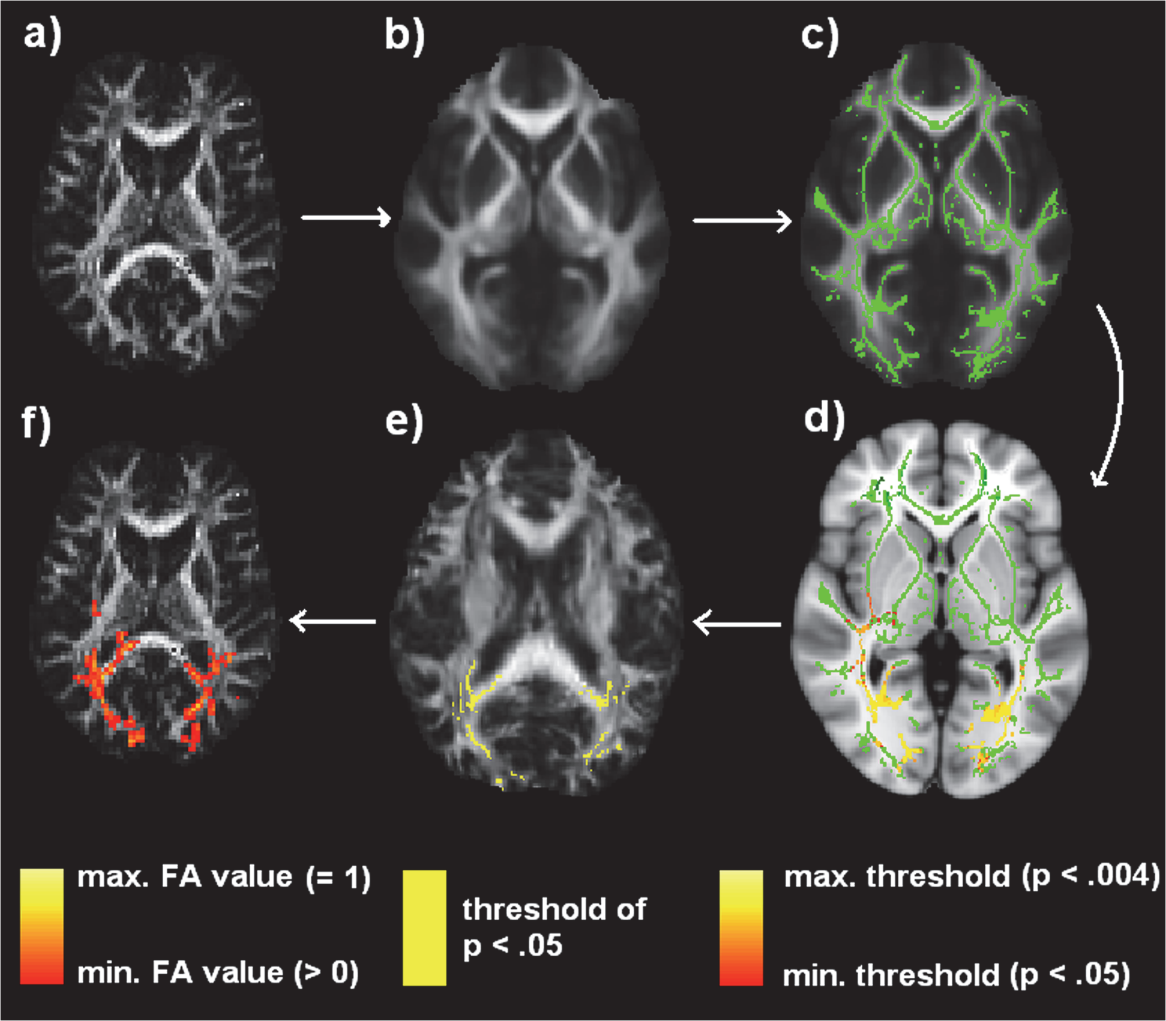
Analysis of the FA data using TBSS (Tract-Based spatial Statistics). a) Individual FA map in native space; b) FA maps of all subjects, transformed into standard 1×1×1 mm MNI152 space and averaged; c) skeletonization of the mean FA image; d) voxel-wise statistics on the skeletonized FA data, indicating significant group differences (yellow: *p* < 0.004; red: *p* < 0.05); e) back-projection from averaged skeleton space to each subject’s standard space for post hoc correlation analyses (color indicates that a given skeleton point matches the threshold of *p* < 0.05 derived from the correct tract center points in each subject), f) back-projection to the space of the native data in FA providing each individual’s exact anatomical locations that contributed to the group effects (yellow: max. FA = 1; red: min. FA > 0, red).

In order to localize diffusion-related changes bound to degenerative processes, the method of”Tract-Based Spatial Statistics” (TBSS, part of FSL software) was applied to the data set, as proposed by Smith and colleagues [57]. TBSS provides voxel-wise statistical analyses based upon nonlinear registration followed by projection onto a tract representation (mean FA skeleton). This approach is characterized by enhanced objectivity and interpretability of multi-subject diffusion imaging data. The following steps of a TBSS analysis were performed (see Fig 1): First, the FA images (Fig 1A) from all subjects (late-blind, normally sighted) were aligned to a pre-identified target FA image by means of nonlinear registration. Afterwards, the aligned FA images were transformed into 1×1×1 mm^3^ MNI152 space by affine registrations. A mean FA image as well as its skeleton was generated from sighted controls as well as the late-blind dataset in MNI152 space (Fig 1B and 1C). The FA maps of individual subjects were projected onto the skeleton. For, finally, comparison of the subgroups (late-blind and sighted individuals), a Threshold-Free Cluster Enhancement (TFCE) statistics [58] for each point on the common skeleton were calculated, using a two sample t-test (Fig 1D). The null distribution of cluster-size statistics was built up over 500 permutations of group membership (TBSS randomize tool, see [59]). The TFCE approach tries to enhance the intensity within cluster-like regions (that exhibit some spatial contiguity) more than background (noise) regions. After TFCE enhancement, thresholding allows for a better discrimination between noise and spatially-extended signals, i.e., voxels with FA differences. Statistical significance was determined at a level of p < 0.05 (family-wise error (FWE) correction for multiple comparisons across space).

**Fig 2:**
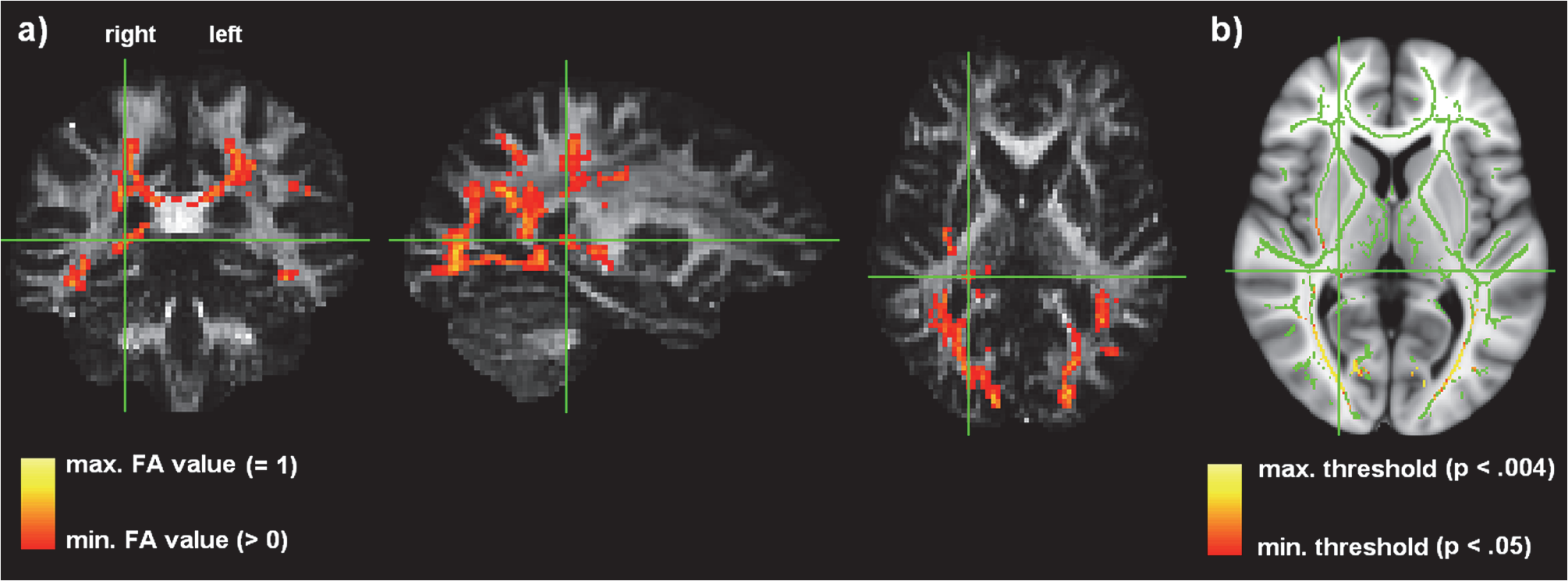
Back-projection to the space of the native data in FA exemplified by a sighted individual (174, see Table 1) (a). In order to obtain an individual’s exact FA value (used for post hoc correlation), the subject’s native anatomy was loaded to find the correct voxel, e.g. the region close to thalamus/Pv (yellow: max. FA = 1; red: min. FA > 0, red), according to (b) the location of the significant cluster resulting from the group comparison (yellow: *p* < 0.004; red: *p* < 0.05).

Post hoc analyses—using SPSS (IBM SPSS Statistics 20)—allowed for the assessment of the significance of any correlations between individual maximal FA values (voxel-level) and behavioral performance of ultra-fast speech comprehension across subjects. However, creating a skeletonized mean FA image based upon TBSS (as it was done in the whole-brain/skeleton analysis) cannot guarantee that a significant voxel contains data from the same part of the same white matter tract from each subject. Furthermore, individual FA characteristics should, on the one hand, be consistent with respect to voxels located within the same white matter tract and should, on the other hand, indicate differences within the white matter tract that are highly relevant to the individual capabilities of understanding ultra-fast speech. Therefore, post hoc correlation analyses relied on back-projections onto the native space of each subject, shedding light onto individuals’ FA data points contributing to the group effect. Thus, the skeleton-space blobs—being significantly different between the two groups of subjects (sighted and late-blind)—were projected back onto the space of the original subjects’ data (Fig 1E and 1F). The back-projection of skeleton-space voxels, first, allows for checking whether the skeleton points had been derived from the correct tract-center points in all subjects. Second, this approach confirmed that the exact subjects’ FA values had been derived rather than being smoothed off by interpolation (Fig 2). The correlation analyses were corrected for two comparisons (Bonferroni Holm), taking into account the number of regions pertaining to our hypotheses: skill-related structural changes (FA value increase) in the (i) right-hemisphere visual pathway and (ii) ipsilateral region close to Pv. The data obtained from the control group are only presented in a descriptive manner, because inter-individual variation in behavioral performance, ranging from 4 to 21% only (except for the single trained individual), was too low for further correlational analyses. Furthermore, partial correlation analyses were performed to show the differential effects of age, onset and duration of blindness, and behavioral performance. A priori, normal distribution of the FA data was tested (Shapiro-Wilk-test).

## Results

### Behavioral data

A normal distribution could be determined for chronological age, blindness onset, and the duration of blindness within the late-blind subject group. Behavioral performance of ultra-fast speech comprehension showed a normal distribution in normally sighted participants (excluding the trained subject), but not in late-blind individuals (*p* <. 039). Furthermore, chronological age in the normally sighted controls did not display normal distribution (*p* <. 024). The highly trained sighted individual was considered an outlier and, therefore, not included in the normally sighted control group. Finally, correlations between behavioral performance, on the one hand, and chronological age, blindness onset, as well as duration of blindness, on the other, were calculated within the late-blind group, using the Spearman test.

The groups of late-blind and sighted participants are characterized by a comparable mean age (Mann-Whitney-U-test: *p* = .229). Auditory comprehension of ultra-fast speech utterances (16 syl/s)—in terms of the percentage of correctly reproduced words outside the scanner—extended in late-blind listeners from 0 to 93% (mean = 51.47%, *SD* ± 29.90) since not all the individuals with vision loss had subjected themselves to a training of accelerated spoken language comprehension. In unskilled sighted individuals, the performance level consistently fell below 21% (Table 1; mean = 10.64%, *SD* ± 5.68). As already mentioned, the dataset included a single sighted participant who had trained ultra-fast speech comprehension—across a time-interval of approximately six months (see [46]). Most notably, this individual achieved a performance level of 88% at a speech rate of 16 syl/s (Table 1). Thus, late-blind and normally sighted groups (without the trained sighted subject) showed significant differences in speech comprehension of ultra-fast test materials (Mann-Whitney-U-test: *p* <. 005). Furthermore, the enhanced perceptual skills of late-blind listeners showed a tendency for a negative correlation with the age of onset of vision loss (two-tailed Spearman: *ρ* = -.508, *p* = .053) as well as with chronological age (two-tailed Spearman: *ρ* = -.483, *p* = .068). Additionally, a significant correlation between chronological age and age of onset of vision loss (two-tailed Pearson: *r* = .725, *p* <. 002) as well as duration of blindness emerged (two-tailed Pearson: *r* = -.870, *p* <. 000).

### Alterations in fractional anisotropy (FA)

**Fig 3:**
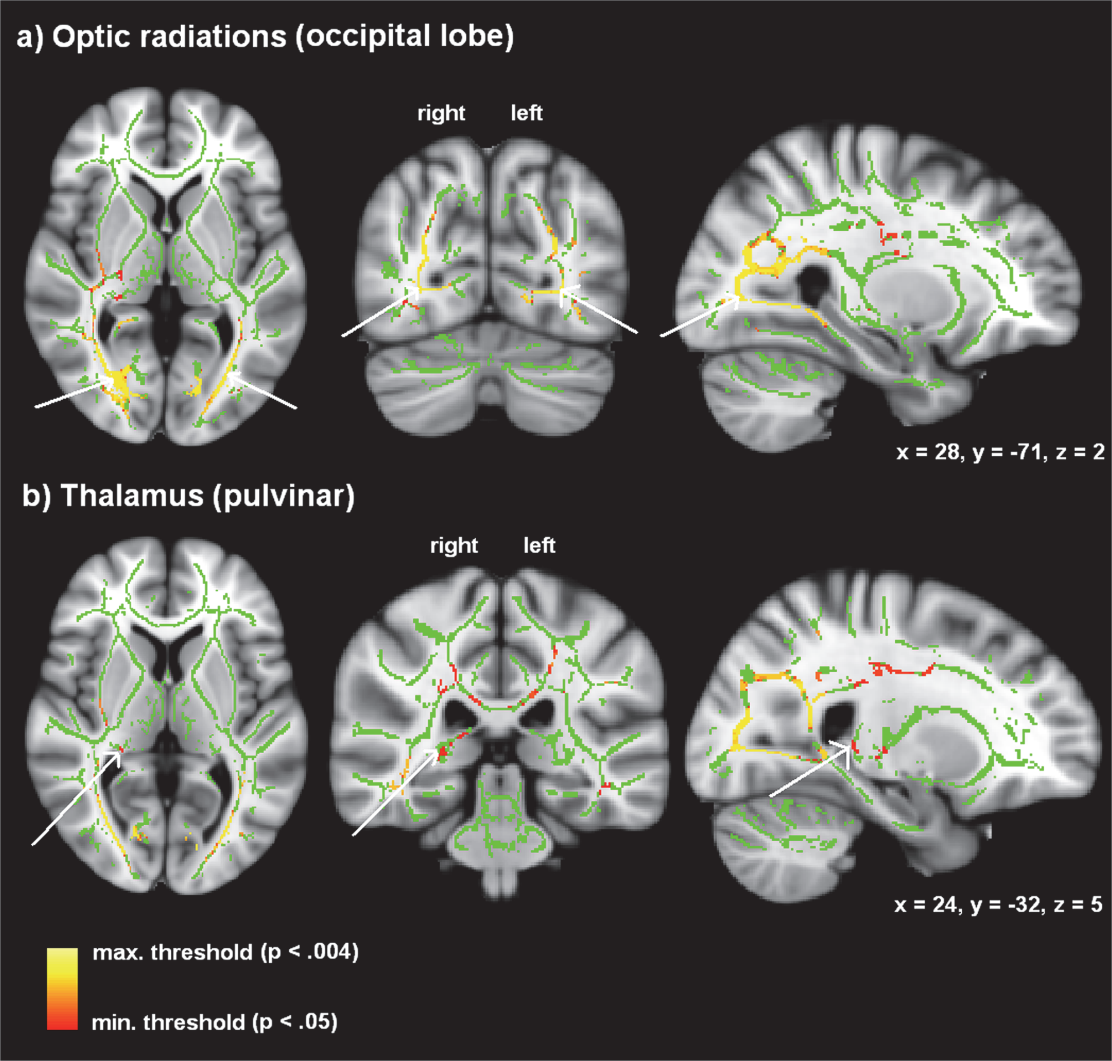
Clusters with reduced FA values in the late-blind compared to the sighted control group (yellow: *p* < 0.004; red: *p* < 0.05) overlaid on the mean FA skeleton map (green). Significant effects were found within (a) the optic radiation of both hemispheres and (b) the right-hemisphere dorsal thalamus/Pv (threshold: TFCE statistic, family-wise error (FWE) corrected *p* < 0.05).

As compared to their sighted controls, late-blind subjects showed significantly reduced FA values of the optic radiation within the occipital lobe of either hemisphere (Table 2, Fig 3, and S1 Table). These regions encompass fibers of the inferior fronto-occipital/longitudinal fasciculus. Furthermore, significant FA reduction of the left-hemisphere optic radiation at the level of the temporal lobe, including the sagittal stratum, could be found in late-blind subjects (Table 2 and S1 Table). In addition, an area adjacent to the right-hemisphere dorsal thalamus/Pv, including fibers of the anterior thalamic radiation, displayed reduced FA values (Table 2, Fig 3 and S1 Table) in the late-blind group (compared with sighted controls). This subcortical thalamic region is supposed to be connected with temporal, occipital, posterior parietal, and prefrontal regions (FSL 4.1.1 atlas tool, Oxford Thalamic Connectivity Probability atlas; see S1 Table). Finally, the corticospinal tracts of the late-blind subjects were characterized by a significant FA reduction at the level of the midbrain (Table 2 and S1 Table). The contrast “late-blind > sighted”, by contrast, did not yield any significant effects.

**Table 2:** Peak coordinates (MNI), cluster extent, and anatomical regions of the contrast “sighted > late-blind” (two sample *t*-test).

**Anatomical locations of the peak**	**Side**	**Coordinates**			**Number of voxels**	**Significance level**
Optic radiation (occipital lobe); inferior longitudinal / fronto-occipital fasciculus	right	28	-71	2	9039	*p* < 0.004
Optic radiation (occipital lobe); inferior longitudinal / fronto-occipital fasciculus	left	-28	-75	2	4015	*p* < 0.004
Optic radiation (temporal lobe); Sagittal stratum, inferior longitudinal / fronto-occipital fasciculus	left	-39	-39	-11	97	*p* < 0.044
Thalamus (pulvinar)	right	24	-32	5	90	*p* < 0.044
Corticospinal tracts (midbrain)	right	16	-13	-8	70	*p* < 0.048

Results were exposed at a threshold of *p* < 0.05, family-wise error (FWE) corrected (for further descriptions of anatomical locations see S1 Table).

### Post hoc correlation analyses

The FA data from all the regions with significant alterations—bilateral optic radiation, right-hemisphere thalamus/Pv, contralateral sagittal stratum, and cortico spinal tracts (see above)—showed a normal distribution. First, a partial correlation analysis—controlled for onset, duration, and age—was conducted in order to clarify whether FA values were correlated with the performance. Second, the correlation between onset and FA values was computed—controlled for performance, age, and duration—in order to determine the independent contribution of vision loss onset to changes in FA values. Third, we investigated the correlation between duration and FA values while controlling for age, onset, and performance to evaluate the independent contribution of duration to changes in FA values. Fourth, the correlation between age and FA values was evaluated while controlling for performance, onset, and duration in order to assess the independent contribution of age to changes in FA values. Although the behavioral performance of ultra-fast speech comprehension did not show normal distribution (see above, Behavioral data), its residuals (linear regression between FA and performance with onset, duration, and age as control variables) were normally distributed.

**Fig 4:**
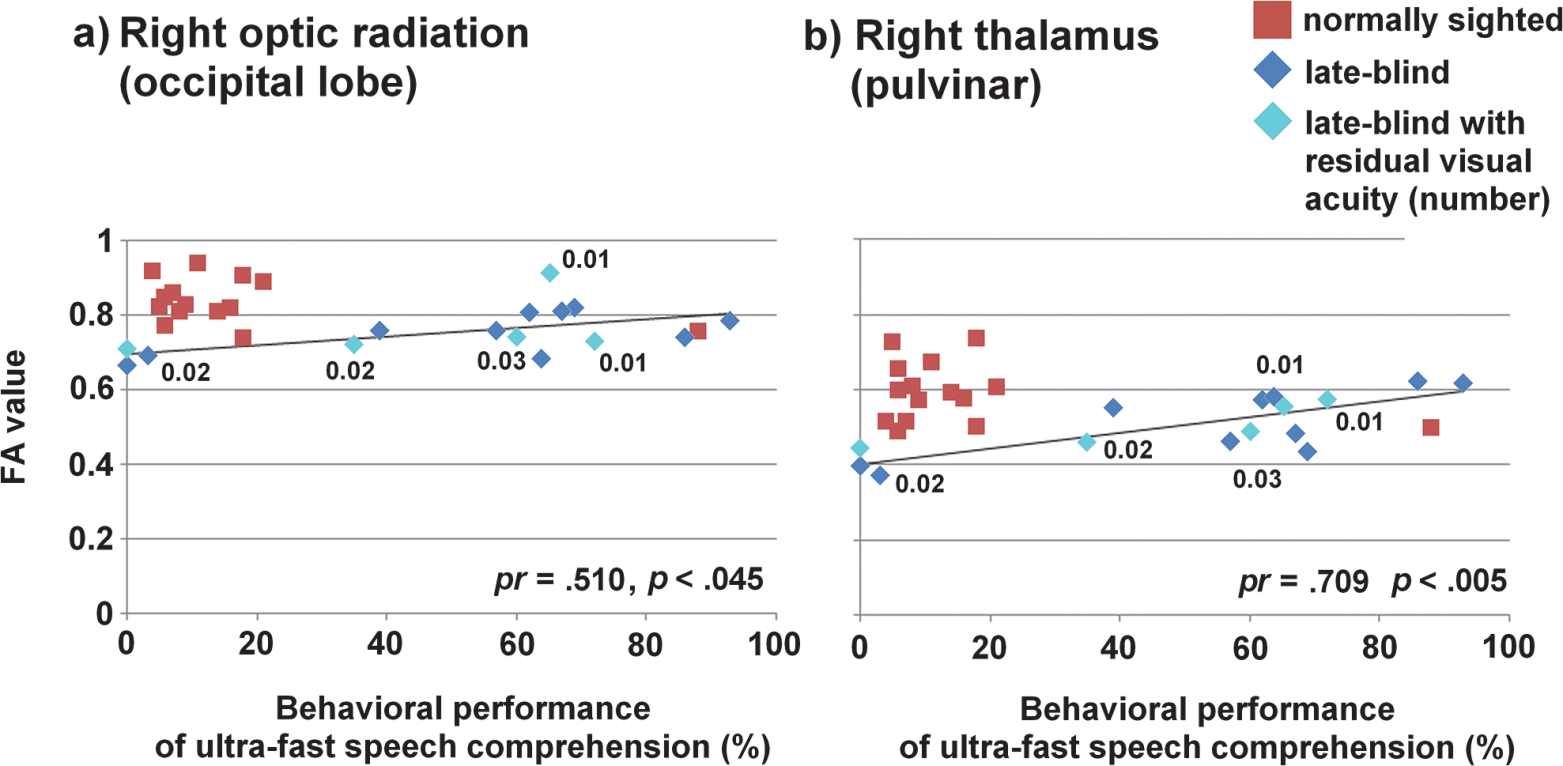
Scatter plots of FA values against the individual behavioral performance of understanding ultra-fast speech. Regression lines, partial correlation coefficients, and significance levels refer to the late-blind group (blue diamonds) only, sighted controls (red squares) are only plotted for descriptive comparison. Values of visual acuity referred to late-blind individuals with residual visual perception (aqua diamonds).

Fig 4 plots the FA values obtained from the right-hemisphere optic radiation (occipital lobe, Fig 4A) and ipsilateral thalamus/Pv (Fig 4B)—referred to the third hypothesis of the present study—against behavioral performance of ultra-fast speech comprehension. The level of statistical significance was Bonferroni Holm corrected for two tests resulting in a threshold of, first, *p* <. 025 and, second, *p* <. 05 (one-tailed tested, because positive correlations between performance and FA values were expected for Pv and the optic radiation of the right hemisphere). After controlling for age, onset, and duration, significant correlations between FA values of the right-hemisphere Pv, the right occipital (positive correlation) and left temporal (negative correlation) part of the optic radiations (latter was not included in the hypothesis and Bonferroni correction) and the performance of understanding ultra-fast speech survived in the late-blind group (partial correlation coefficient (*pr*) = .709, *p* <. 005 for right Pv; *pr* = .510, *p* <. 045 for right occipital optic radiation; *pr* = -.817, *p* <. 001 for left temporal optic radiation). No significant partial correlations were found between the duration (controlling for performance, onset, age), onset (controlling for performance, duration, age), or age (controlling for performance, onset, duration) and FA values within these areas. Thus, in the present late-blind group a linear relationship between behavioral performance and FA values could be observed after excluding the effects of age, onset, and duration of blindness.

Regarding behavioral performance, duration and onset of blindness, chronological age, and FA values (bilateral occipital optic radiations, left temporal optic radiations, right dorsal thalamus/Pv, corticospinal tracts), no significant differences between late-blind individuals with very low (visual acuity between 0.01 and 0.03, *n* = 5) and without residual vision (visual acuity = 0, *n* = 10) could be found (Mann-Whitney-U-test: *p* > 0.1).

## Discussion

### Degeneration of fiber tracts in late-blind individuals

Significantly reduced FA values could be detected along the optic radiation at either side, including the fronto-occipital pathways (inferior fronto-occipital/longitudinal fasciculus) in the group of late-blind individuals. So far, subjects with a delayed onset of vision loss have rarely been investigated by means of structural MRI methods—as compared to early-blind individuals. By and large, the available data are in accordance with the findings of the present study. Two recent investigations reported, e.g., lower FA values of both optic radiations in late-blind individuals, but also in individuals suffering from early vision loss [6–7]. Using tensor-based morphometry, furthermore, Lepore and colleagues [11] found a diminished volume of primary and secondary visual cortices both in late- and early-onset blindness (versus sighted controls each). Another study, indeed, failed to document any notable FA reductions at the level of the optic radiations in late-blind individuals [60]. It must be taken into account, however, that the small sample size (*n* = 6) may have hampered the detection of significant effects. By contrast, Park and colleagues [12] again observed structural changes of primary visual cortex both in early- and late-blind individuals: Both subject groups displayed a reduced surface area concomitant, however, with either enhanced (early blindness) or reduced cortical thickness (late blindness). These data could be later replicated [14, 61].

In addition to the optic chiasm and radiations, voxel-based morphometric measurements have revealed a decrease of gray matter volume of early visual areas (BA 17/18) [4, 9]. However, FA reduction of the optic radiations seems to be more pronounced in late- than early-blind subjects [6], suggesting different underlying mechanisms. Additionally, significant FA reduction and reduced white matter volume could be documented in the geniculo-calcarine tracts [1, 3]. By contrast, Zhang and colleagues [7] reported FA reduction of the optic nerve fibers, but not in the geniculo-calcarine tracts of late-blind participants compared with normally sighted controls. Although the inferior fronto-occipital connections were found uncompromised [3], significant reduction of mean FA values along the whole fiber tract could be observed in early-blind (versus sighted) individuals [2]. In addition to structural changes of the optic radiation, the present study detected reduced FA values at the level of the right-hemisphere dorsal thalamus/Pv, including the anterior thalamic radiation. Preceding investigations in early-blind individuals had found alterations of Pv and its projections to the associative visual areas [9]. Data obtained both from rhesus monkeys [62] and humans [63] suggest Pv to project to multiple cortical areas, including frontal, parietal, temporal, occipital and cingulate cortex, and subcortical structures such as the superior colliculus and amygdala (see [64], for a review). At the functional level, Pv has been assumed to play a role in, e.g., synchronization of auditory and visual information [65] and/or visual change detection [66]. So far, structural alterations of Pv have been documented exclusively in early-blind individuals [9, 67]. The present investigation revealed late-onset blindness also to affect this subcortical structure which supports visual processing in sighted subjects—though significant changes were restricted to the right hemisphere (at the left side, these effects emerged at an uncorrected level only). In line with previous findings [62–63], the present data of the dorsal thalamus/Pv displayed probable connectivity, on the one hand, to temporal and occipital areas and, on the other, to parietal and prefrontal regions. Thus, dorsal thalamus/Pv might serve as an interface linking the visual system to various cortical brain areas. This notion is in line with that thalamo-cortical transmission balances in a dynamic fashion bottom-up and top-down factors [68].

Beyond optic radiations and dorsal thalamus/Pv, the late-blind group of the present study showed (versus sighted controls) reduced FA values within the left-hemisphere sagittal stratum. This region encompasses fibers of the inferior longitudinal fasciculus (= a tract extending from the occipital cortex towards the anterior temporal lobe) and optic radiation. The sagittal stratum is assumed to connect areas of the occipito-temporal cortices [69–70]. However, FA reduction within this region seems to depend on increased capabilities of ultra-fast speech comprehension (see below) rather than blindness-induced degeneration.

Finally, significant FA reduction was also found within the corticospinal tracts at the level of the midbrain. By contrast, previous studies had reported increased FA values of these pathways in early-blind subjects as compared to sighted controls [4, 71]. Yu and colleagues [71] suggested these changes to reflect enhanced motor activities during sensitive developmental periods (childhood). Wang and colleagues [6] documented an increase of the FA values of the corticospinal tracts in late-blind subjects as well, suggesting neuroplastic changes due to demands on the performance of routine activities. These divergent findings (increased versus decreased FA values) might reflect differences in compensatory strategies, e.g., the extent to which sensorimotor substitute visually guided motor coordination. Neuroplastic changes of the corticospinal tracts have also been observed, e.g., in sighted individuals after extensive piano practice during sensitive developmental periods (childhood) [72]. It is, thus, conceivable that diminished motor activities following vision loss might give rise to regressive alterations of the efferent projections of motor cortex.

To summarize, the late-blind individuals of the present study showed more or less the same distributional pattern of white matter changes—in terms of FA reduction—as the early-blind subjects of preceding investigations, including the optic radiations and thalamus/Pv. In addition, FA reduction was found to affect occipito-temporal fibers as well as the corticospinal tracts.

### Skill-dependent cross-modal reorganization

The late-blind individuals—varying in their capabilities of ultra-fast speech comprehension—showed a significant positive correlation (post hoc) between FA values and perceptual capabilities within right-hemispheric optic radiation (occipital lobe) and dorsal thalamus/Pv of the same side. Whereas considerable evidence points at experience- or task-related reorganization of visual cortex functions in early- and late-blind humans [25, 27, 29, 73], skill-related structural changes of fiber tracts have rarely been documented so far. For instance, reduced FA values of white matter structures beneath left-hemisphere temporo-parietal regions have been found to covary with reading skills in adult subjects and also to differ in reading-impaired and literate children [74–75]. Madden and colleagues [76] reported a moderate correlation between the reaction time in a visual target detection task and FA of the splenium of the corpus callosum. Additionally, average FA of the corticospinal tracts was found significantly increased in early-blind men, but not early-blind women—related, presumably, to changes of motor experience during sensitive development periods [71]. In the context of an explicit training task, previous studies found training of a complex visuo-motor skill, i.e., juggling, to cause structural changes in gray matter [77] as well as FA alterations of white matter tracts [78]. Furthermore, cortical thickness of the occipital cortex appears to predict auditory capabilities in both early- and late-blind individuals [14]. Finally, a recent study by Anurova and colleagues [79] reported significant negative correlations between the cortical thickness of occipital areas in early-blind individuals and functional hemodynamic activation of those regions during sound-localization or pitch-identification tasks. Moreover, one of the few studies investigating the relationship between white matter alterations and behavioral capabilities (auditory/tactile discrimination tasks) reported a strong relationship between a marker of myelin content and behavioral performance in early- and/or late-blind individuals [15]. Taken together, the present data provide—based upon ultra-fast speech perception as an experimental paradigm—further support for the suggestion that training of behavioral traits can be associated with structural changes—in terms of FA values—of distinct white matter areas.

The significant correlation between FA and behavioral performance was found restricted to the right hemisphere—as expected against the background of our preceding functional imaging studies. Previous studies on ultra-fast speech comprehension in late-blind listeners clearly point at a significant covariance between right-, but not left-hemisphere V1 activation and the capability of understanding time-compressed verbal utterances [45]. Although ultra-fast speech comprehension must recruit language processing mechanisms and, thus, might be expected to elicit left-hemisphere activation [29–30, 38, 47], the strong temporal constraints of ultra-fast speech perception might invoke right-hemisphere mechanisms of prosody processing. The syllabic structure of speech—an aspect of prosody relevant to the timing and relative weighting of segmental phonetic information [80]—provides a temporal grid for the generation of articulation-related speech representations in frontal cortex during perception. In line with the Asymmetric Sampling Model as a mechanism underlying hemispheric cortical lateralization effects [81–82], it has been shown that syllabic amplitude modulation of the speech envelope is predominantly represented at the level of the right hemisphere [83–85]. In other words, ultra-fast speech comprehension requires rapid temporal processing with respect to both (i) suprasegmental information (e.g., tracking metric contours of syllable onset streams)—assigned as a function of the right hemisphere—as well as (ii) segmental phonological information as a function of the left hemisphere. In light of our previous functional imaging studies [45–46] and the present structural investigation, ultra-fast speech perception seems to push the suprasegmental rather than the segmental speech encoding components to their limits, in line with findings that the bottleneck of time-compressed speech processing is located in superordinate structures of language processing rather than left-hemisphere auditory phonetic resolution [86]. Against this background, we hypothesized [49] that a right-hemisphere dominant syllabic timing mechanism should be—in a not yet fully specified manner—linked via SMA to a left-dominant network of phonological processing during speech encoding.

The only sighted subject who had so far undergone training in ultra-fast speech comprehension did not exhibit any increased FA values in comparison to the untrained sighted individuals, neither within the optic radiation nor the thalamus/Pv. Obviously, thus, this single participant had not been able to recruit the visual system in order to improve ultra-fast speech comprehension. However, it remains an open question whether involvement of the optic radiation and thalamus/Pv during ultra-fast speech processing is exclusively bound to the group of late-blind participants.

Interestingly, the FA values of the left hemisphere optic radiation at the level of the temporal lobe (sagittal stratum) were negatively correlated with the behavioral measures of ultra-fast speech comprehension. Rather than just reflecting degeneration due to vision loss, FA reduction within the sagittal stratum, thus, seems to be somehow related to the late-blind subjects' training of ultra-fast speech perception. This region encompasses fibers of the inferior longitudinal fasciculus (= a tract extending from the occipital cortex towards the anterior temporal lobe) and optic radiation (see above) and, thus, might connect areas of the occipito-temporal cortices [69–70]. As a consequence, the practice of listening to ultra-fast speech might influence cortico-cortical temporo-occipital connectivity. Speculatively, left-hemisphere phonological strategies being reported to recruit the left-hemisphere occipital cortex in blind individuals [29, 40], might be reduced in order to make the right-hemisphere prosodic strategy much more efficient.

Regarding the interpretation that can be drawn from the positive correlation between FA and performance, listening to ultra-fast speech might be assumed to affect the central-visual system in terms of revitalization or preservation of occipital white matter structures. However, given that cross-modal plastic phenomena are likely to occur on a similar time scale as the deprivation-related white matter atrophy ([87] for review), the inverse causality cannot be excluded, i.e., that preserved white matter tracts directly facilitate ultra-fast speech comprehension. However, a previous functional imaging study [46] investigating late-blind subjects (suffering from blindness for a long time) who were trained in ultra-fast speech comprehension showed that (i) the skill could be acquired during a period of a few months and (ii) hemodynamic activation of right visual cortex increased after training. Thus, regarding the short training period in comparison to blindness onset dating back several years, a development in parallel can be excluded in these subjects. Thus, although intra-individual structural data of the training study are not yet available, these data might provide further support for a hypothesis of practice- and skill-dependent reorganization.

### Future research goals

The structural changes regarding axonal organization or myelination might be further evaluated by other diffusion-related data, for example based on diffusion tensor eigenvalues (axial/radial) or mean diffusivity. In the present study, the eigenvalues (λ _1_, λ _2_, λ _3_) indicating diffusivity parallel (λ _1_) or perpendicular to the principle axis (λ _2_, λ _3_) of a fiber bundle were not separately analyzed. Further, the white matter tracts that showed significant FA reduction have crossing-fibers along their tracts. Thus, white matter fractional anisotropy is significantly underestimated with a single diffusion tensor model at crossing-fiber regions. As part of a future research goal, we, therefore, aim the calculation of axial, radial, and mean diffusivity as well as tract-specific FA analyses corrected for the effects of crossing-fiber geometry at those regions [88].

## Conclusions

Blindness-induced degeneration, i.e., reduced FA values, could be documented along the optic radiation adjacent to the visual cortex at either side and in the region of the right-hemisphere dorsal thalamus/Pv in a group of late-blind subjects (versus sighted controls). Furthermore, these participants displayed a significant positive correlation between the level of ultra-fast speech comprehension—in terms of the percentage of correctly reproduced items in a repetition task—and the FA values calculated for right-hemisphere optic radiation and ipsilateral thalamic/Pv—an observation indicating experience-related structural neuroplasticity within the respective areas. Late-blind subjects, thus, seem to be able to enhance auditory speech processing which might at least partially associated with preservation or revitalization of those components of the visual system. The observed structural changes are, finally, consistent with previous functional imaging data obtained from a comparable subject group, suggesting that, especially, right-hemisphere visual areas are involved in ultra-fast speech processing at an already early, i.e., signal-related processing stage (e.g., [45]).

## Supporting Information

S1 Table Peak coordinates (MNI) and anatomical regions of the contrast “sighted > late-blind” (two sample *t*-test). Results were exposed at a threshold of *p* < 0.05, family wise error (FWE) corrected.
(DOCX)
