# Supplementary material for: Experience-Related Structural Changes of Degenerated Occipital White Matter in Late-Blind Humans – A Diffusion Tensor Imaging Study
Source: PLoS One. 2015 Apr 1;10(4):e0122863. doi: 10.1371/journal.pone.0122863 (PMC4382192; doi:10.1371/journal.pone.0122863)
Supplement: S1 Table — Results were exposed at a threshold of p < 0.05, family wise error (FWE) corrected. (DOCX) [file pone.0122863.s001.docx]

| **Table S1.** Peak coordinates (MNI) and anatomical regions of the contrast “sighted > late-blind” (two sample *t*-test). Results were exposed at a threshold of *p* < 0.05, family wise error (FWE) corrected. | | | |
| --- | --- | --- | --- |
| **Anatomical locations of the peak regarding distinct Atlases:**  **^1^Harvard-Oxford Cortical Structural Atlas**  **^2^ Harvard-Oxford Subcortical Structural Atlas**  **^3^ JHU ICBM-DTI-81 White-Matter Labels**  **^4^ JHU White-Matter Tractography Atlas**  **^5^Juelich Histological Atlas**  **^6^ MNI Structural Atlas**  **^7^ Oxford Thalamic Connectivity Probability Atlas**  **^8^Talairach Daemon Labels** | **Coordinates** | | |
|  | **X** | **Y** | **Z** |
| ^1^1% occipital fusiform gyrus, 1% lingual gyrus  ^2^99% right cerebral white matter, 2% right cerebral cortex  ^4^50% right inferior fronto-occipital fasciculus, 26% forceps major, 21% right inferior longitudinal fasciculus  ^5^59% right white matter optic radiation, 27% white matter callosal body, 16% gray matter visual cortex V1, BA17  ^6^5% occipital lobe, 2% parietal lobe  ^8^right cerebrum, occipital lobe, lingual gyrus, gray matter | 28 | -71 | 2 |
|  |  |  |  |
| ^1^1% intracalcarine cortex  ^2^98% left cerebral white matter  ^4^29% left inferior fronto-occipital fasciculus, 21% left inferior longitudinal fasciculus, 13% forceps major  ^5^51% left white matter optic radiation, 24% white matter callosal body  ^6^35% occipital lobe  ^8^left cerebrum, occipital lobe, sub-gyral, white matter | -28 | -75 | 2 |
|  |  |  |  |
| ^1^11% temporal fusiform cortex, posterior division, 2% inferior temporal gyrus, posterior division, 1% temporal occipital fusiform cortex, 1% inferior temporal gyrus, temporo-occipital part  ^2^77% left cerebral white matter, 15% left cerebral cortex  ^3^sagittal stratum (include left inferior longitudinal fasciculus and left inferior fronto-occipital fasciculus)  ^4^29% left inferior longitudinal fasciculus, 13% left inferior fronto-occipital fasciculus  ^5^69% left white matter optic radiation, 24% white matter callosal body  ^6^4% temporal lobe  ^8^left cerebrum, temporal lobe, sub-gyral, white matter | -39 | -39 | -11 |
|  |  |  |  |
| ^2^78% right cerebral white matter, 16% right thalamus, 3% right hippocampus, 2% right cerebral cortex , 1% right lateral ventricle  ^3^fornix (cres) / stria terminalis (can not be resolved with current resolution)  ^4^5% anterior thalamic radiation  ^5^15% white matter fornix, 10% right gray matter hippocampus cornu ammonis, 7% right gray matter hippocampus dentate gyrus  ^6^2% thalamus  ^7^54% temporal, 54% occipital, 33% posterior parietal, 3% pre-frontal  ^8^right cerebrum, sub-lobar, thalamus, gray matter, pulvinar | 24 | -32 | 5 |
|  |  |  |  |
| ^2^56% right cerebral white matter, 4% right cerebral cortex , 3% right pallidum  ^3^right cerebral peduncle  ^4^ right 8% corticospinal tract  ^5^62% right white matter corticospinal tract, 3% right gray matter hippocampus hippocampal-amygdaloid transition area, 1% right gray matter hippocampus dentate gyrus  ^8^right brainstem, midbrain | 16 | -13 | -8 |
| Abbreviation: BA = Brodman area. |  |  |  |
